# Evaluating of Gene Expression Alteration after Garlic Consumption, Analyzing through Bioinformatics Approach 

**DOI:** 10.22037/ijpr.2020.112409.13744

**Published:** 2021

**Authors:** Somayeh Esmaeili, Mohammad Rostami-Nejad, Mostafa Rezaei-Tavirani, Farshad Okhovatian, Mohammad Mehdi Zadeh-Esmaeel, Zahra Razzagh, Alireza Ahmadzadeh, Reza Vafaee

**Affiliations:** a *Traditional Medicine and Materia Medica Research Center, School of Traditional Medicine, Shahid Beheshti University of Medical Sciences, Tehran, Iran. *; b *Gastroenterology and Liver Diseases Research Center, Research Institute for Gastroenterology and Liver Diseases, Shahid Beheshti University of Medical Sciences, Tehran, Iran. *; c *Proteomics Research Center, Faculty of Paramedical Sciences, Shahid Beheshti University of Medical Sciences, Tehran, Iran. *; d *Physiotherapy Research Center, Shahid Beheshti University of Medical Sciences, Tehran, Iran. *; e *Skin Research Center, Shahid Beheshti University of Medical Sciences, Tehran, Iran. *; f *Laser Application in Medical Sciences Research Center, Shahid Beheshti University of Medical Sciences, Tehran, Iran. *; g *Proteomics Research Center, Shahid Beheshti University of Medical Sciences, Tehran, Iran.*

**Keywords:** Garlic, Spleen, Network analysis, Gene, Biomarker

## Abstract

Molecular study of garlic as a popular food ingredient could better understand its health benefits such as immunological effects. For this aim, effects of garlic on the spleen and possible side effects including oxidative stress increment, the molecular mechanism is investigated through network analysis of differentially expressed genes in the treatment of garlic. Protein-protein interaction (PPI) network analysis of spleen gene expression profile of *Mus musculus *(8-week old male C57BL/6J mice) in garlic treatments from a microarray study with the code of GSE10344 was analyzed via GEO2R software. Furthermore, Cytoscape V 3.7.1 was applied to construct and analyze a network of up- and down-regulated genes. The differentially expressed genes (DEGs) were analyzed via the CluePedia plugin of Cytoscape to determine expression patterns. After the identification of central nodes, an action map was created. A total of 77 DEGs were achieved which were including 40 up-regulated and 37 Down-regulated. The centrality analysis of the network indicated that *Vcan*, *Lamb1*, and *Ltbp1* are hubs and *Glra1*, *Wdr17*, *Nefl*, and *Becn1* are bottlenecks. Mutual regulatory connections between hubs and *Alb* and *App* (as two non-queried hubs) were determined. The findings indicate that garlic effect on the spleen and its mechanism may be involved mostly with App dysregulation.

## Introduction

Diet plays an indispensable role in human health. Historically, garlic (*Allium sativum *L.) has been popular for its potential benefits and therapeutic influence worldly. Aside from being a health promotor, it can reduce the risk of many diseases. It can lower the chance of cancer, microbial, viral and fungal infection, cardiovascular diseases, and diabetes mellitus (1, 2). The main constituents of garlic are alliin, allicin, diallyl disulfide, ajoene and ascorbic acid (3). The complex chemical foundation of garlic is accounted for its vast health benefits (4). It has traditionally been reported to have expectorant, diaphoretic, disinfectant, and diuretic properties. More recently, it has also been reported that garlic health benefits vary as the genetic content differs. Some of the important known characteristics of garlic are its antioxidant and antimicrobial properties (2). The two most influenced microbes are bacteria *Proteus mirabilis* and Antibiotic-resistant *Escherichia coli* (5). Some of the reported cancer treatments with garlic are bladder (6), pancreatic carcinoma (7), gastric and colon cancers (8). Besides, it is reported that garlic plays role in regulation of immune system function. Since spleen is related to the immune system, garlic consumption can improve its function via cellular and humoral immune response modulation (9). On the other hand, garlic could also have contradictory properties at the same time when prescribed at high doses. It has been reported that high doses of garlic could cause some damages in spleen. Anemia is one of the main problems (10). Molecular studies can be helpful for exploration of mechanism of different kinds of treatments (11, 12). Microarray studies is one of the ways to investigate differentially expressed genes (DEGs) in the treated samples. The DEGs could be important as the contributing elements of action of the applied treatment of interest in the organ. They could consider as biomarkers after sequence of complementary studies. Bioinformatics analysis is also productive in terms of providing more knowledge about the related DEGs in the treated samples. Here, to better understand the valuable effects of garlic on spleen and possible side effects, the molecular mechanism is investigated through protein-protein interaction analysis of differentially expressed genes in the treatment of garlic. The central nodes of the constructed network will be analyzed.

## Experimental

This study investigates the garlic benefits on spleen function by meta-assaying the transcriptome profile of the mentioned organ. Gene Expression Omnibus (GEO) database (https://www.ncbi.nlm.nih.gov/geo/) was selected for our bioinformatics approach (13). There are many gene expression data series about different samples in certain conditions in GEO. Using a platform to obtain data; GPL1261, the data Serie GSE10344 (https://www.ncbi.nlm.nih.gov/geo/query/acc.cgi?acc=GSE10344), including samples as GSM261472-7, the dataset was analyzed through GEO2R, the online software available on GEO. This study was conducted by Akgül B, Lin K, Tu CD in 2008 on spleen gene expression profile of *Mus musculus *(8-week old male C57BL/6J mice) which one group was fed with cellulose and the other with garlic powder diet. The exact conducted procedure was as follows: the first group named as control with three repeats fed with 4% cellulose (14). The second as the treated group also with three repeats fed with a special diet of 4% lyophilized raw garlic powder. The spleen organ of these groups was removed for transcriptome analysis after 15-weeks of diet. As mentioned, the dataset was analyzed with GEO2R, and the *p*-value ≤ 0.05 and fold change (FC) > 2 were considered for differentially expressed genes (DEGs) identification. At first, the GEO2R provides R Script (a formula for analyzing and screening DEGs) and then the further statistical analysis would be carried out with R Studio Software. The DEGs from the top 250 statistically significant ones were determined and then those with gene names were searched against the network constructing software, Cytoscape 3.7.1 and STRING db (15, 16). Prior to the network constructions, CluePedia analyzed the pattern of the expression of the significant DEGs in the whole dataset. This application links to the dataset available in GEO input file and finds the expressions pattern for the queried genes (17). The network centrality analysis was handled by NetworkAnalyzer application based on two important parameters including degree (K) and betweenness centrality (BC). Genes with highest amount of degree and betweenness are considered as hubs and bottlenecks respectively. The hubs and bottlenecks were then screened for their action types in CluePedia software. The statistical criterion for this analysis was based on kappa scoring. All the provided action types by CluePedia including expression, activation, inhibition, catalysis, binding, ptmod, and reaction were analyzed through this plug-in and the kappa score was set to 0.5. 

## Results

**Figure 1:**
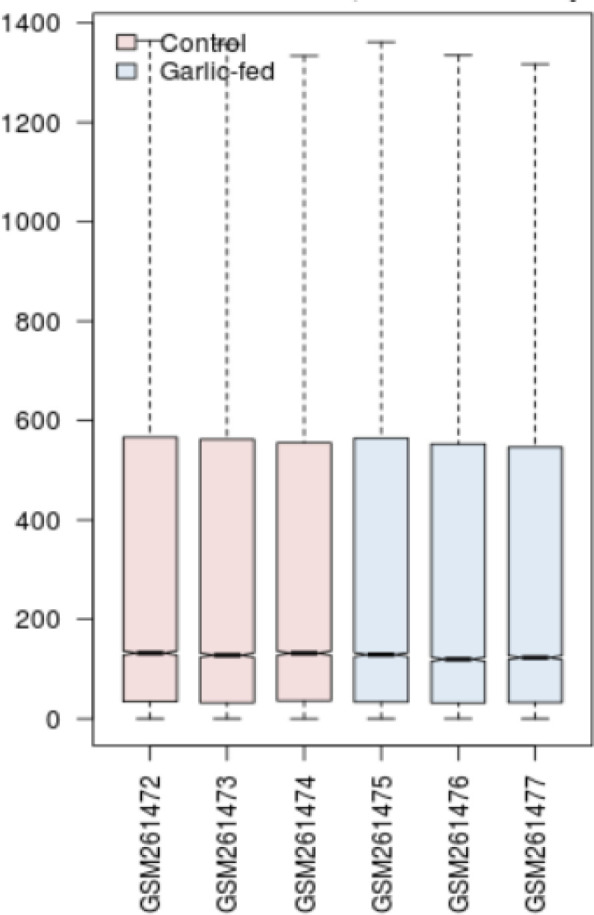
The value distribution of normal-fed and garlic-fed comparison via box-plot analysis. The pink plot indicates control, whereas the blue one shows the garlic-treated samples

A box-plot could be useful to analyze if the groups of samples are comparable in terms of expression. If the samples are median-centered, they are acceptable for comparison. This presentation is illustrated in the Figure 1. As shown in Figure 1, the two upper quarters have a depressed distribution relative to the two other quarters.

**Figure 2:**
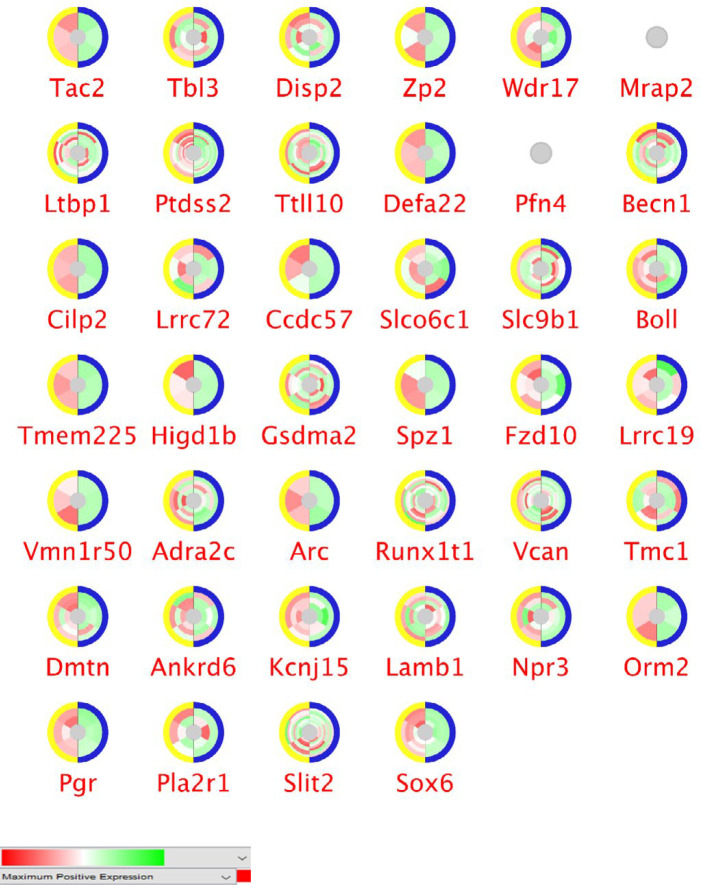
The normalized expression pattern for 40 significant up-regulated genes of the spleen in the treatment with garlic. Around each gene, color scheme changes refer to the values of expressions. Red is the maximum positive expression while green indicates negative expression changes. The white color indicates no expression and the grey color shows missing values in this presentation. The colors of outer circle indicate control and the treated group. The yellow color is the garlic treated group and blue is the control

**Figure 3:**
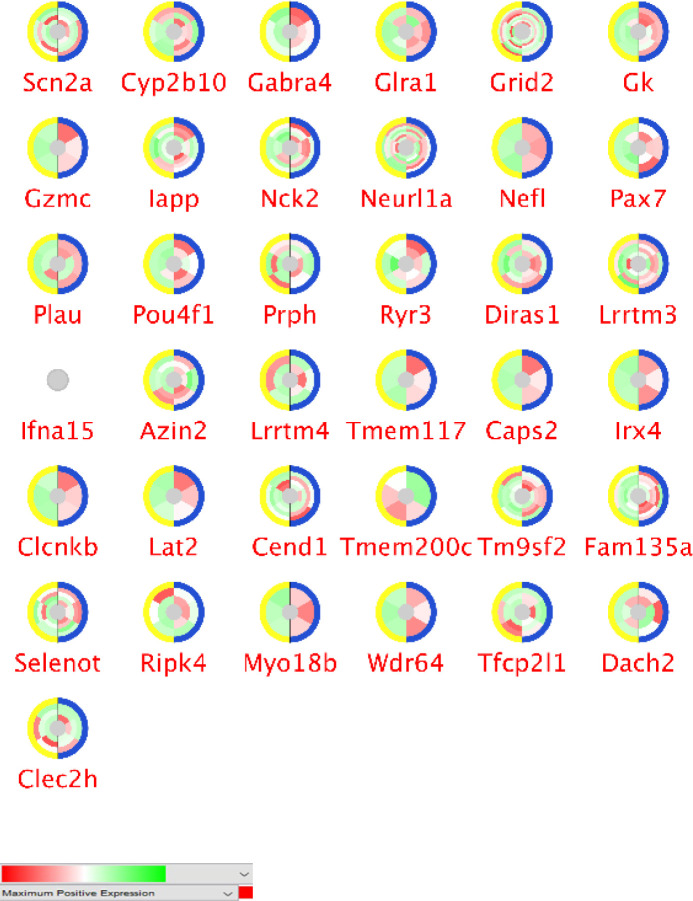
The normalized expression pattern for 37 significant down-regulated genes of the spleen in the treatment with garlic. Around each gene, color scheme changes refer to the values of expressions. Red is the maximum positive expression while green indicates negative expression changes. The white color indicates no expression and the grey color shows missing values in this presentation. The colors of outer circle indicate control and the treated group. The yellow color is the garlic treated group and blue is the control

Differentially expressed genes between cellulose and garlic-fed groups were analyzed with GEO2R. The top 250 significant genes with *p*-value < 0.05 and FC > 2 are gathered for more assessment. The expression profiles of the significant DEGs via CluePedia are shown in Figures 2 and 3. Each circle consists of two splits (control and treated) that each half contains three parts. Each part indicates biological repeats. In a microarray dataset, more than one differentially expressed spot may be available for each gene. The layers of colors range of green and red indicate the number of spots. To consider a gene with significant expression changes in the dataset, at least one spot should be statistically significant in expression alterations. 

In Figures 2 and 3, the expression values are missing for Mrap2 and PFn4 in the up-regulated set and for Ifna15 in the down-regulated set. Most of our genes have more than one spot in the dataset. The expression variation is not equal for all spots. Some genes specify an inhomogeneous pattern of expressions, whereas others are closer in this regard. For instance, Ltbp1 is with more than one spot and does not show a homogenous expression pattern. Contrariwise, Gzmc is with one spot and shows homogenous expression changes. All of the genes are expressed statistically significant in the garlic treated samples as at least one spot with significant DEG is related to them. 

**Figure 4:**
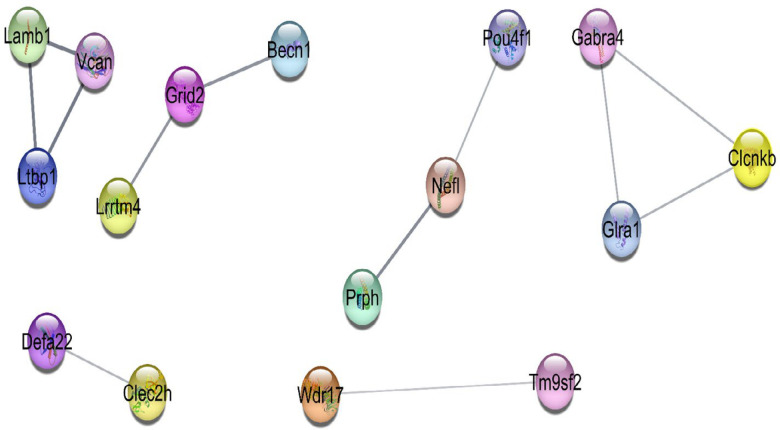
The six connected units of 16 genes in the direct connections. Four connected units with three nodes and the other two components with two nodes. Due to the poor interactions between the queried DEGs, the rest of the DEGs were isolated

Two DEGs networks were constructed; the first one consists of only DEGs to understand their interaction behavior without any additive neighbors. The second one includes DEGs and 50 added neighbors. In the first network (the data is not shown), a number of 77 genes were obtained and only 12 edges between them. A number of 16 genes are in the connected units and the rest remained as individuals nodes (Figure 4). 

The units of connected genes in the first query as shown in Figure 4, are (*Lamb1*, *Vcan*, and *Ltbp1*), (*Prph*, *Nefl*, and *Pou4f1*), (*Glra1*, *Clcnkb*, and *Gabra4*), (*Defa22 *and *Clec2h*), and (*Wdr17* and *Tm9sf*). 

**Figure 5:**
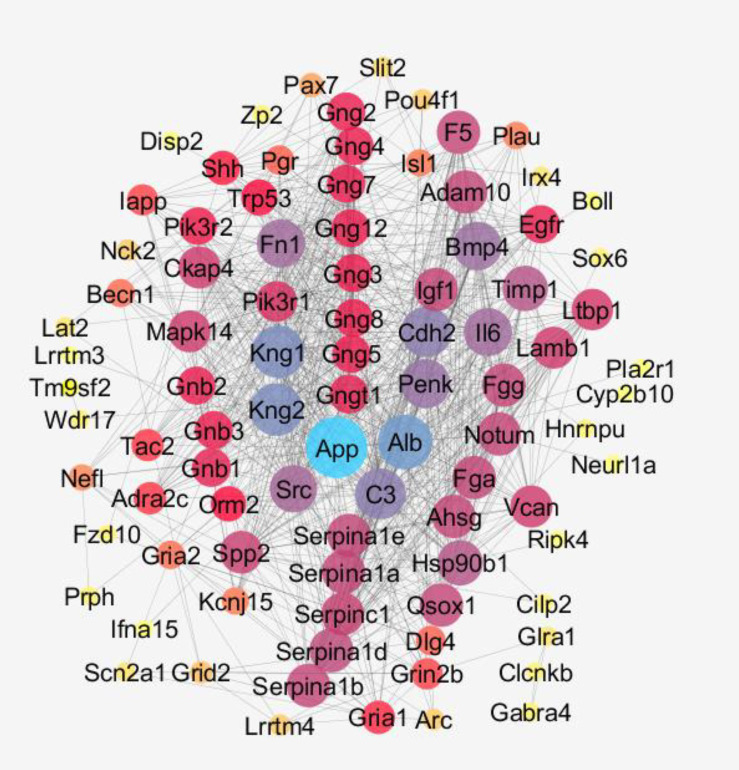
The centrality analysis of the first component of the second network. The most central gene (the potent hub node) is colored blue. The bigger the size and yellow to blue color refer to highest values of degree

The second network consists of 127 nodes and 876 connections. This network contains 41 up-regulated genes and 37 down-regulated ones. A number of 91 genes are in condense connections while the rest are present as individual nodes. The component with 91 genes was extracted for network centrality analysis (Figure 5). As depicted in this figure, the nodes are discriminated based on their impact on making connections with the other nodes. 

In Figure 5, the node with the highest degree value is assigned with light blue color. This gene is App with the degree of 58. The next gene is *Alb* and the degree value for this gene is 48. *App* also has a high value of betweenness. These two genes are considered as hub-bottlenecks of the constructed network of garlic-treated samples. 

The 10% top nodes based on degree value determined as hubs, including *Vcan*, *Lamb1, Ltbp1,* and *Orm2*. The last hub, degree value, is significantly different from the other three top hubs (19 *versus* 28). Therefore, this hub was ignored. Similarly, bottlenecks were identified based on betweenness centrality as; *Glra1*, *Wdr17*, *Nefl*, and *Becn1*. Description of hubs and bottlenecks is extracted from the STRING database and is presented in Table 1. 

In Table 1, seven central DEGs are presented as either hubs or bottlenecks. Out of them, five genes are assigned with positive FC while the rest are with negative FC. Moreover, screening the central genes in Figures 2 and 3, specify that they are present as multi-spots in the dataset. 

**Figure 6:**
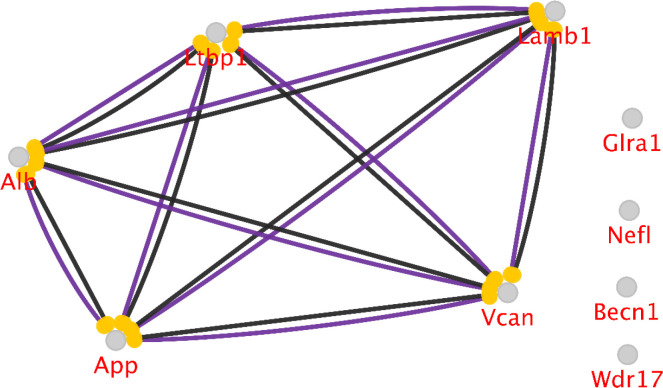
The action view of seven searched genes of hubs and bottlenecks with the addition of the most central genes of *App* and *Alb*. The actions between three hubs and two hub-bottlenecks are present as catalysis (purple) and reaction (black). The rest of the nodes did not show any action type. Other types of actions, including activation, inhibition, and expression, were not found

The action map of seven hubs and bottlenecks with the addition of the most central genes of *App* and *Alb* (which belong to the added neighbors) is shown in the Figure 6. *Glra1*, *Nefl*, *Becn1*, and *Wdr17* are not involved in the network. Therefore, they do not play any regulatory role on the other elements of the action map. In the action type analysis, all the provided action types with the kappa score of 0.5 were queried for hubs and bottlenecks. However, only two types of actions presented which were between the *Ltbp1*, *Lamb1*, and *Vcan* as the hubs. 

## Discussion

A deeper understanding of garlic benefits consumption could be accessible through molecular analysis such as complementary analysis of array studies. One of these ways is to screen the expression profile of datasets containing transcriptome data. One of the organs that may be influenced by garlic intake is the spleen. Spleen has novel contributions to homeostatic actions in our body. Interestingly, garlic can boost this key role of the spleen. For example, garlic can promote the T helper cytokines balance in response to many kinds of diseases (18). Meanwhile, high doses of garlic could also be toxic in our body, such as oxidative stress increment (19). Researchers have shown that allicin, the main component of garlic could reduce spine inflammation due to the reduction of some important inflammatory factors such as *TNF-a* in an animal model. Additionally, allicin could inhibit HLA-B2704 protein expression without effect on the expression of the *HLA-B2704* gene (20)

 Here, the control and garlic-fed groups were compared in terms of a difference in gene expression via network analysis. The sample values are median-centered. Therefore, they are proper for further research. 

The GEO2R analyzed the groups and through R studio Software, a number of 77 genes were assigned as DEGs among the 250 top statistically significant ones. Numbers of 40 genes were up-regulated while 37 ones were down-regulated. Two networks from these DEGs were constructed, in which the first one was only consist of DEGs, while the second network also contains the neighbor genes. There are some genes in connection in the first network, while most of the genes were obtained as separate nodes. The behavior of connected units of genes was screened in the second network in terms of centrality values. The centrality evaluation of the second network indicated that some genes are further essential in the structure of the map. These genes are *App* (Amyloid-beta A4 protein) and *Alb* (Serum albumin) that are the most vital hub-bottlenecks of the network of garlic treatments. These genes are not from the DEGs and they came from the neighbors. There are four spots of the *App* gene available in the studied dataset in which none was with statistical significance in differential expression in the garlic treatment. Two of these spots showed no statistically significant up-regulation with the fold of 2 and 1.5. As mentioned earlier, this gene is also the most central gene in the network of garlic treatment.

As analyzing further, it can be realized that some of the DEGs could also have centrality properties. Seven genes are divided into two groups of hubs and bottlenecks. The hubs, including *Vcan* (versican), *Lamb1* (laminin B1), and *Ltbp1* (latent transforming growth factor-beta binding protein 1), while possessing high values of degree, they do not have any significant bottleneck values. All these genes are highly up-regulated in the garlic-treated sample. *Vcan* shows the highest value of the differential expression. These genes also exist as a connected unit in the first network query. The second group, bottlenecks; however, are not present as condensed units in the first network but rather disperse elements in the units. These nodes are *Glra1*, *Wdr17*, *Nefl*, and *Becn1*. Among them, *Glral* has the highest value of betweenness centrality. Degree value as like betweenness is corresponded to dispersed nodes however *Becn1* expresses the highest amount of degree. Therefore, the central DEGs are not considered as hub-bottlenecks at the same time. Regarding the expression changes of these bottlenecks, while Glra1 (glycine receptor, alpha 1 subunit) and Nefl (neurofilament, light polypeptide) show down-regulation in the treatment of garlic, Wdr17 (WD repeat domain 17) and Becn1 (beclin 1, autophagy-related) indicate up-regulations. Apparently, up-regulation is dominant among the central genes in the treatment of garlic. To better recognize the central DEGs roles, the action type between these nodes and the hub-bottlenecks (App and Alb) were analyzed by the CluePedia platform. The analysis also suggested the key position of the DE hubs and their tight interactions with the hub-bottlenecks in the garlic treated network since catalysis and ptmod actions are present. The text mining of the hub-bottlenecks and central DE elements can provide more knowledge of their position in garlic mechanisms of action. 

Based on the critical role of App and Alb in the control of the central DEGs, it can be concluded that expression change of these two genes is an important process in the effect of garlic consumption. Considering the importance of App in Alzheimer’s disease (AD) promotion, in the following part, more details of expression change of App gene under the effect of garlic feeding is discussed: 

 App gene, as the most central gene in the network of garlic treatment, is an important gene in relation to AD pathogenicity. This gene is also expressed in other organs such as spleen as well but with the lower amounts (21). This gene showed no statistical significance in expression difference in the spleen of mice fed with garlic. However, two spots of this gene indicated highlighted up-regulations. On the other hand, regardless of the prominent beneficial contribution of spleen in immune responses to many diseases, this organ could sometimes have harmful consequences in the body organs such as the brain. Brain-spleen inflammatory coupling is phenomena that indicate the important connection of immune activity of spleen and brain function. In brain injury, the production of spleen cytokines increases dramatically and results in brain inflammation (22). In addition, spleen production such as Syk (spleen tyrosine kinase) has been reported to have a regulatory effect in (AD). This correlation is by promotion of Aβ gathering and hyperphosphorylation of Tau in brain which results in AD development (23). As it is reported garlic has been suggested as a neuroprotective food due to its anti-amyloidogenic, anti-inflammatory, and anti-tangle properties (24, 25). As mentioned earlier, application of high dosage of garlic could also have adverse effects on the spleen. One of which is the increase of oxidative stress. This process is accounted for Syk activation as well, which is responsible for AD formation (26). There is a possibility that, the higher dosage of garlic treatments may be accompany with induction of significant up-regulation of App gene. However, the potential role of this gene in underlying mechanism of garlic on spleen has yet to analysis furtherer. However this investigation was focused on effect of garlic on spleen it seems that the gene expression alteration occurs in the other organs as well as spleen. 

**Table1:** List of hubs and bottlenecks; Description is extracted from STRING db and is summarized. BC, D, and FC refer to betweenness centrality, degree, and fold change, respectively

**R**	**Name**	**description**	**BC**	**D**	**FC**
1	*Vcan*	Chondroitin sulfate proteoglycan core protein 2; May play a role in intercellular signaling and connecting cells with the extracellular matrix. May take part in the regulation of cell motility, growth and differentiation. It binds hyaluronic acid.	0.001	29	13
2	*Lamb1*	Laminin-10 subunit beta; Binding to cells via a high-affinity receptor, laminin is thought to mediate the attachment, migration and organization of cells into tissues during embryonic development by interacting with other extracellular matrix components. It involves in the organization of the laminar architecture of the cerebral cortex (By similarity). It is probably required for the integrity of the basement membrane/glia limitans that serves as an anchor point for the endfeet of radial glial cells and a physical barrier to migrating neurons (By similarity). Radial glial cells play a central role in cerebral cortical development, where they act both as the proliferative unit of the cerebral cortex and a scaffold for neurons migrating toward the pial surface (By similarity).	0	29	3.5
3	*Ltbp1*	Latent transforming growth factor-beta binding protein 1; May be involved in the assembly, secretion and targeting of TGFB1 to sites at which it is stored and/or activated. May have a structural role in the extracellular matrix (ECM); Belongs to the LTBP family.	0	28	8.5
4	*Glra1*	Glycine receptor strychnine-binding subunit; Glycine receptors are ligand-gated chloride channels. Channel opening is triggered by extracellular glycine. Channel opening is also triggered by taurine and beta-alanine (By similarity). Plays an important role in the down-regulation of neuronal excitability. Contributes to the generation of inhibitory postsynaptic currents. Channel activity is potentiated by ethanol. Potentiation of channel activity by intoxicating levels of ethanol contributes to the sedative effects of ethanol.	0.044	3	-12.8
5	*Wdr17*	WD repeat domain 17	0.022	2	3
6	*Nefl*	Neurofilament, light polypeptide; Neurofilaments usually contain three intermediate filament proteins: L, M, and H which are involved in the maintenance of neuronal caliber.	0.008	10	-11
7	*Becn1*	Coiled-coil myosin-like BCL2-interacting protein; Plays a central role in autophagy. Play a role in multiple membrane trafficking pathways: PI3KC3-C1 is involved in the initiation of autophagosomes and PI3KC3-C2 in the maturation of autophagosomes and endocytosis. Involved in regulation of degradative endocytic trafficking and required for the abscission step in cytokinesis, probably in the context of PI3KC3-C2 (By similarity). Involved in endocytosis, including endosome formation in neuronal cells. May play a role in antiviral host defense (By similarity).	0.007	12	8.6

## Conclusion

The finding implies that garlic dietary influence on the human body could be a dose-dependent substance. It is suggested that the possible linkage between garlic effect on spleen and App gene contribution in this light to be studied. 
